# Long-term risk prediction after major lower limb amputation: 1-year results of the PERCEIVE study

**DOI:** 10.1093/bjsopen/zrad135

**Published:** 2024-01-24

**Authors:** Brenig Llwyd Gwilym, Philip Pallmann, Cherry-Ann Waldron, Emma Thomas-Jones, Sarah Milosevic, Lucy Brookes-Howell, Debbie Harris, Ian Massey, Jo Burton, Phillippa Stewart, Katie Samuel, Sian Jones, David Cox, Annie Clothier, Hayley Prout, Adrian Edwards, Christopher P Twine, David Charles Bosanquet, Vascular and Endovascular Research Network (VERN) and the PERCEIVE study group, Aminder Singh, Athanasios Saratzis, Brenig Llwyd Gwilym, David Charles Bosanquet, George Dovell, Graeme Keith Ambler, Joseph Shalhoub, Louise Hitchman, Matthew Machin, Nikesh Dattani, Panagiota Birmpili, Rachael Forsythe, Robert Blair, Ruth Benson, Ryan Preece, Sandip Nandhra, Sarah Onida, Amy Campbell, Anna Celnik, Bryce Renwick, Jolene Moore, Karen Duncan, Martin Gannon, Mary Duguid, Patrice Forget, Dhafer Kamal, Mahmoud Tolba, Martin Maresch, Mohamed Hatem, Mohamed Kabis, Ahmed Shalan, Hannah Travers, Maciej Juszczak, Mohammed Elsabbagh, Nikesh Dattani, António Pereira-Neves, João Rocha-Neves, José Teixeira, Eric Lim, Khaleel Hamdulay, Oliver Lyons, Ashraf Azer, Chris T Francis, Khalid Elsayed, Ragai Makar, Shady Zaki, Tamer Ghatwary-Tantawy, Devender Mittapalli, Hashem Barakat, Jessica Taylor, Ross Melvin, Samantha Veal, Anna Pachi, Antonia Skotsimara, Chrisostomos Maltezos, Christiana Anastasiadou, Efstratia Baili, George Kastrisios, Konstantinos Maltezos, Athanasios Saratzis, Badri Vijaynagar, Elizabeth Montague-Johnstone, Euan Bright, Kirsty Stewart, Rahul Velineni, Simon Lau, Will King, Christina Papadimitriou, Christos Karkos, Maria Mitka, Emily Chan, George Smith, Aditya Vijay, Anita Eseenam Agbeko, Joachim Amoako, Joseph Shalhoub, Matthew Machin, Afroditi Antoniou, Konstantinos Roditis, Nikolaos Bessias, Paraskevi Tsiantoula, Theofanis Papas, Vasileios Papaioannou, Fiona Goodchild, George Dovell, Claire Dawkins, James Rammell, Sandip Nandhra, Andrea Mingoli, Gioia Brachini, Paolo Sapienza, Pierfrancesco Lapolla, Alan Meldrum, Keith Hussey, Lara Dearie, Manoj Nair, Andrew Duncan, Bryony Webb, Stefan Klimach, Francesca Guest, Tom Hardy, Annie Clothier, Luke Hopkins, Ummul Contractor, Dominic Pang, Li En Tan, Meghan Hallatt, Olivia McBride, Rachael Forsythe, Ben Thurston, Jacqueline Wong, Nishath Altaf, Oliver Ash, Amandeep Grewal, Matthew Popplewell, Steven Jones, Bethany Wardle, Christopher Twine, Francesca Heigberg-Gibbons, Graeme Ambler, Kit Lam, Natalie Condie, Mustafa Musajee, Prakash Saha, Sanjay Patel, Stephen Black, Thomas Hayes, Ankur Chawla, Anthony Feghali, Asad Choudhry, Eric Hammond, Michael Costanza, Palma Shaw, Ronald Zerna Encalada, Scott Surowiec, Craig Cadwallader, Philipa Clayton, Ruth Benson, Isabelle Van Herzeele, Lina Vermeir, Mia Geenens, Nathalie Moreels, Sybille Geers, Arkadiusz Jawien, Tomasz Arentewicz, Emmanouil Tavlas, Nikolaos Kontopodis, Stella Lioudaki, Vasiliki Nyktari, Abdulhakin Ibrahim, Alexander Oberhuber, Jana Neu, Teresa Nierhoff, Konstantinos Moulakakis, Konstantinos Nikolakopoulos, Spyros Papadoulas, Stavros Kakkos, Mario D’Oria, Sandro Lepidi, Danielle Lowry, Frances Kent, Setthasorn Ooi, Benjamin Patterson, Daniel Urriza Rodriguez, Gareth F Williams, Ghadeer Hesham Elrefaey, Ibrahim Enemosah, Kamran A Gaba, Simon Williams, Elizabeth Suthers, Manar Khashram, Odette Hart, Sinead Gormley, Stephen French, Hytham K S Hamid

**Affiliations:** School of Medicine, Cardiff University, Cardiff, UK; Gwent Vascular Institute, Royal Gwent Hospital, Aneurin Bevan University Health Board, Newport, UK; Centre for Trials Research, Cardiff University, Cardiff, UK; Centre for Trials Research, Cardiff University, Cardiff, UK; Centre for Trials Research, Cardiff University, Cardiff, UK; Centre for Trials Research, Cardiff University, Cardiff, UK; Centre for Trials Research, Cardiff University, Cardiff, UK; Centre for Trials Research, Cardiff University, Cardiff, UK; Artificial Limb and Appliance Centre, Rookwood Hospital, Cardiff and Vale University Health Board, Cardiff, UK; Artificial Limb and Appliance Centre, Rookwood Hospital, Cardiff and Vale University Health Board, Cardiff, UK; Artificial Limb and Appliance Centre, Rookwood Hospital, Cardiff and Vale University Health Board, Cardiff, UK; Department of Anaesthesia, North Bristol NHS Trust, Bristol, UK; C/O INVOLVE Health and Care Research Wales, Cardiff, UK; C/O INVOLVE Health and Care Research Wales, Cardiff, UK; School of Medicine, Cardiff University, Cardiff, UK; Centre for Trials Research, Cardiff University, Cardiff, UK; Division of Population Medicine, Cardiff University, Cardiff, UK; Bristol, Bath and Weston Vascular Network, North Bristol NHS Trust, Southmead Hospital, Bristol, UK; School of Medicine, Cardiff University, Cardiff, UK

## Abstract

**Background:**

Decision-making when considering major lower limb amputation is complex and requires individualized outcome estimation. It is unknown how accurate healthcare professionals or relevant outcome prediction tools are at predicting outcomes at 1-year after major lower limb amputation.

**Methods:**

An international, multicentre prospective observational study evaluating healthcare professional accuracy in predicting outcomes 1 year after major lower limb amputation and evaluation of relevant outcome prediction tools identified in a systematic search of the literature was undertaken. Observed outcomes at 1 year were compared with: healthcare professionals’ preoperative predictions of death (surgeons and anaesthetists), major lower limb amputation revision (surgeons) and ambulation (surgeons, specialist physiotherapists and vascular nurse practitioners); and probabilities calculated from relevant outcome prediction tools.

**Results:**

A total of 537 patients and 2244 healthcare professional predictions of outcomes were included. Surgeons and anaesthetists had acceptable discrimination (C-statistic = 0.715), calibration and overall performance (Brier score = 0.200) when predicting 1-year death, but performed worse when predicting major lower limb amputation revision and ambulation (C-statistics = 0.627 and 0.662 respectively). Healthcare professionals overestimated the death and major lower limb amputation revision risks. Consultants outperformed trainees, especially when predicting ambulation. Allied healthcare professionals marginally outperformed surgeons in predicting ambulation. Two outcome prediction tools (C-statistics = 0.755 and 0.717, Brier scores = 0.158 and 0.178) outperformed healthcare professionals’ discrimination, calibration and overall performance in predicting death. Two outcome prediction tools for ambulation (C-statistics = 0.688 and 0.667) marginally outperformed healthcare professionals.

**Conclusion:**

There is uncertainty in predicting 1-year outcomes following major lower limb amputation. Different professional groups performed comparably in this study. Two outcome prediction tools for death and two for ambulation outperformed healthcare professionals and may support shared decision-making.

## Teaser

This manuscript reports the 1-year results from the PERCEIVE study. We aimed to evaluate the accuracy of healthcare professionals (surgeons, anaesthetists and allied healthcare professionals) in predicting outcomes at 1 day following a major lower limb amputation and evaluate the accuracy of relevant amputation-specific risk prediction tools in a prospective cohort of patients undergoing major lower limb amputation. Our findings show that healthcare professionals were reasonably accurate in predicting the risk of death but were poor at predicting revision surgery and ambulation. We demonstrate that two death outcome prediction tools outperformed healthcare professionals. Two outcome prediction tools aiming to predict ambulation performed better than healthcare professionals, but estimations were still poor.

## Introduction

Decision-making when considering major lower limb amputation (MLLA) is complex. Patients who undergo MLLA face several risks such as wound infection/breakdown^1,2^, hospital readmission^2,3^, MLLA revision surgery^2,4,5^, medical morbidities (for example myocardial infarction or lower respiratory tract infection)^5^, psychological morbidity^6–8^, social isolation^9–11^ and death^2–5,12^.

Weighing up risks and benefits of options with patients and/or relatives and carers is part of shared decision-making^13^. Surgeons typically are worse at predicting longer-term outcomes than short-term outcomes for surgery in general, and their performance relative to outcome prediction tools varies^14^. Long-term (1-year after surgery) outcome prediction models specific to MLLA are available, but validation studies are typically lacking^15^. It is unknown how accurate healthcare professionals, or outcome prediction models, are at predicting the longer-term outcomes following MLLA.

The objectives of PrEdiction of Risk and Communication of outcomE following major lower limb amputation: a collaboratIVE study (PERCEIVE) were to determine how accurate healthcare professionals and relevant outcome prediction tools were at predicting outcomes following MLLA. The early results of PERCEIVE have shown that healthcare professionals were more accurate than most outcome prediction models in predicting death, morbidity risk and MLLA revision at 30 days following MLLA^16^.

This article reports the long-term results of PERCEIVE, which aimed to examine how accurate healthcare professionals and relevant outcome prediction tools were at predicting death, MLLA revision and ambulation at 1 year following MLLA. Additional objectives were to explore whether healthcare professional characteristics (profession and seniority) or patient characteristics (COVID-19 status and indication for MLLA) influenced prediction accuracy.

## Methods

A protocol describing the PERCEIVE quantitative study methods has been published^17^.

### Design and setting

This article reports an international, multicentre, prospective observational cohort study in accordance with the ‘Strengthening the Reporting of Observational Studies in Epidemiology’ (STROBE) statement^18^.

The study launched on 1 October 2020. Centres began patient recruitment in a ‘staggered start’ manner once necessary local approvals were obtained. Baseline and operative data collection on patients undergoing MLLA at centres providing an elective and/or emergency vascular service continued until 1 May 2021. One-year follow up was completed on 1 May 2022.

The study followed a collaborative research model. The PERCEIVE study team designed and delivered the study with support from the Vascular and Endovascular Research Network (VERN)^19^. Local study teams at each participating centre comprised a lead and up to seven other healthcare professionals/students.

### Inclusion and exclusion criteria

Adults undergoing MLLA for acute/chronic limb threatening ischaemia and/or diabetes mellitus were suitable for inclusion. Patients having revision MLLA surgery or having MLLA for other indications (such as cancer or trauma) were excluded.

### Patient identification, data capture and quality control

Local study teams prospectively identified patients satisfying the inclusion/exclusion criteria, and healthcare professionals eligible to provide predictions of outcomes. Different healthcare professional groups were only asked to predict outcomes appropriate to their expertise/role: death (surgeons and anaesthetists), MLLA revision (surgeons), and ambulation (surgeons and allied healthcare professionals (vascular nurse practitioners and specialist physiotherapists)). Healthcare professionals were only eligible to give predictions if they were sufficiently familiar with the patient and would be happy to do so for the specific patient in usual clinical practice.

Predictions of risk of death and MLLA revision were made before surgery and were recorded on a standardized visual analogue scale ranging from 0 to 100% circulated to each centre or given verbally as a percentage. Ambulation was predicted before surgery using the following categories: bedbound/chairbound; able to use a wheelchair only; able to use a prosthesis to stand/transfer only (The Special Interest Group in Amputee Medicine (SIGAM) grade B); able to use a prosthesis for ambulating (SIGAM grade C or greater)^20^. Multiple healthcare professionals could give predictions for the same patient but did so independently. Patients without any healthcare professional predictions were still eligible for inclusion for the evaluation of outcome prediction tools.

Baseline demographic, operative and outcome data were collected from healthcare records. A protocol circulated to each centre prior to data collection included instructions and clear definitions of any baseline data/outcomes that could be subjectively interpreted. Patients were not directly contacted at any point. Data were managed using the Research Electronic Data Capture (REDCap) system^21^. Participation in data validation was mandatory for all centres, as per previous collaborative studies^22–25^. This included a team member, not involved in initial data capture, recapturing 25% of datapoints for 20% of the caseload from each centre to evaluate accuracy. Validation also included evaluating case ascertainment, where a team member compared the number of cases included in PERCEIVE with the number of MLLAs performed at the centre who would be eligible for inclusion.

### Objectives

The primary objectives were to determine the accuracy of healthcare professionals in predicting death, MLLA revision and ambulation 1 year after MLLA. MLLA revision was defined as a return to theatre for revision of soft tissue or re-amputation at the same or higher level. Ambulation was categorized as: bedbound/chairbound; able to use prosthesis to stand/transfer only (SIGAM mobility grade B); able to use prosthesis to ambulate (SIGAM mobility grade C or higher)^20^. Accuracy evaluation included measures of discrimination, calibration and overall performance (Brier score) as appropriate^16,17,26^.

Secondary objectives were to determine the accuracy of relevant outcome prediction tools, and the observed incidence of death, MLLA revision and ambulation after surgery.

### Updated systematic search for outcome prediction tools

A recent systematic review identified six outcome prediction tools designed to predict outcomes at 1 year following MLLA^15^. To ensure that any relevant outcome prediction tools published after this review was conducted were not missed, an updated systematic search of the MEDLINE and Embase databases was conducted using the same search terms and inclusion/exclusion criteria applied to a limited date range (5 March 2020 to 2 August 2022). Following de-duplication, the titles and abstracts of resultant articles were screened for eligibility. This updated review was used to inform the choice of outcome prediction tools for validation in PERCEIVE.

### Statistical methods

Continuous baseline demographic and operative data were presented as median and range. Categorical baseline, operative and outcome data were presented as frequencies and percentages.

Discrimination was quantified using receiver operating characteristic curves and C-statistics (that is area under the curve (AUC)). C-statistic results were interpreted as: 0.5 no better than chance; 1 perfect^27^. C-statistics were presented with 95% De-Long confidence intervals (c.i.)^28^. Whenever possible, calibration was evaluated primarily with visual inspection of the calibration curve (a scatter plot of the predicted and observed outcome)^29^. Several different methods of quantifying calibration were also calculated and reported: calibration slope (perfect value = 1), intercept (perfect value = 0) and calibration-in-the-large (perfect value = 1). Overall performance was evaluated using the Brier score which considers discrimination and calibration (range 0–1, perfect value = 0). Since ambulation was defined and predicted as a multicategory outcome, a multiclass AUC was calculated which is an average of all ‘pairwise’ AUCs^30^, and no calibration curves were generated as no probabilities were predicted. Where possible, outcome prediction models were evaluated by using their regression equation to calculate the predicted probability.

Primary analyses of outcome prediction models used case-wise deletion, and analyses using ‘worst-case’ imputation of missing variables served as sensitivity analyses. To account for multiple predictions per patient, ‘per-prediction’ analyses were conducted, as opposed to ‘per-patient’ analyses (where the multiple predictions are averaged).

All analyses were performed using the statistical programming environment ‘R’ (version 4.1.0) with add-on packages ‘pROC’ (version 1.18.0), ‘rms’ (version 6.2-0) and ‘ggplot2’ (version 3.3.5)^31–34^.

### Subgroup analyses

Planned subgroup analyses were performed by: healthcare professional profession and seniority, healthcare professionals who reported using an outcome prediction tool to inform predictions routinely and those who do not, and indication for procedure. Subgroup analyses based on COVID-19 status were undertaken for both healthcare professionals and outcome prediction tools.

### Approvals

Aneurin Bevan University Health Board approved the study as a service evaluation. Prior to data collection UK centres needed local audit or research and development department approval; centres outside of the UK required local (for example institutional review board or ethics) approval.

## Results

### Demographic and procedural details

**Fig. 1:**
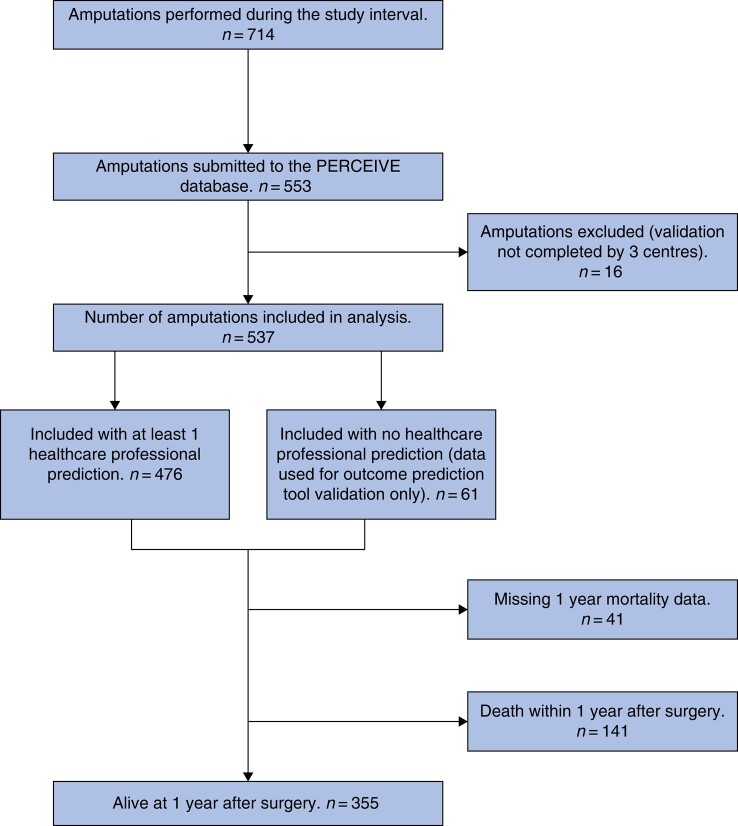
Flow diagram of patients in the study PERCEIVE, PrEdiction of Risk and Communication of outcomE following major lower limb amputation: a collaboratIVE study.

A flow diagram representing the pathway through the study is shown in *Fig. 1*. The full demographic and procedural details for the cohort have already been published (*Supplementary Material S1*)^16^; a summary is presented below.

Data validation confirmed accuracy to be >95% and the overall case ascertainment to be 77.5%. Data on 537 patients who underwent MLLA at 38 centres were included in analyses, 476 (88.6%) of which had at least one preoperative prediction of an outcome by a healthcare professional. Most patients (*N* = 361) had their procedures at 1 of 22 UK centres, and 176 patients at 1 of 16 centres outside the UK. The median age was 68 years (range 19–94), 433 (80.6%) were male, 271 (50.5%) underwent below-knee amputation, 248 (46.2%) underwent above-knee amputation and 17 (3.2%) underwent through-knee amputation (1 missing, 0.2%).

### Existing outcome prediction tools identification

None of the 559 papers screened in the updated systematic review satisfied the inclusion/exclusion criteria (*Supplementary Material S2*). The six risk prediction tools evaluated in this study were those identified in a recent systematic review that predicted outcomes at 1 year following MLLA (*Table 1*)^15^. The variables required for each of these outcome prediction tools are shown in *Supplementary Material S3*.

**Table 1:** Demographic details of the studies to develop outcome prediction tools that were evaluated

First author and year	Country	Prospective/retrospective	Single centre/multicentre	Registry data	Outcome predicted	Number of patients
Norvell *et al* 2019^35^	USA	Retrospective	Multicentre	Yes	Death	5028
Kim *et al* 2021^36^	New Zealand	Retrospective	Multicentre	Yes	Death	21 597
Campbell *et al* 2019^37^	New Zealand	Retrospective	Multicentre	Yes	Death	270 105
Czerniecki *et al* 2019^38^	USA	Retrospective	Multicentre	Yes	MLLA revision	5260
Czerniecki *et al* 2017^39^	USA	Prospective	Multicentre	Yes	Ambulation	157
Bowrey *et al* 2019^40^	UK	Retrospective	Single centre	No	Ambulation	350

### Observed outcomes

A total of 496 patients had complete 1-year death data; 141 of 496 (28.4%) patients died within 1 year of their procedure; 25 of 141 deaths (17.7%) were attributable to COVID-19; 53 of 496 patients (10.7%) underwent MLLA revision within 1 year. There were 260 of 495 (52.5%) patients who were bedbound/chairbound, 54 of 495 (10.9%) who were able to use a prosthesis to stand/transfer and 125 of 495 (25.3%) who were able to use a prosthesis to ambulate following MLLA.

### Predictive performance of healthcare professionals and outcome prediction tools

*Table 2* demonstrates the predictive accuracy of all healthcare professionals and outcome prediction tools in predicting death, MLLA revision and ambulation.

**Table 2:** Performance metrics of healthcare professionals and outcome prediction tools in predicting death, MLLA revision and ambulation at 1 year after MLLA

Death					
Predictor	C-statistic (95% c.i.)	Calibration slope	Calibration intercept	Calibration-in-the-large	Brier score
**Healthcare professionals**					
All healthcare professionals	0.715 (0.679,0.750)	0.549	−0.533	1.198	0.200
Consultant surgeons	0.725 (0.662,0.788)	0.543	−0.627	1.240	0.189
Consultant anaesthetists	0.720 (0.654,0.786)	0.576	−0.399	1.147	0.211
Trainee surgeons	0.716 (0.646,0.786)	0.570	−0.647	1.295	0.195
Trainee anaesthetists	0.680 (0.579,0.781)	0.486	−0.312	1.025	0.220
**Outcome prediction tools**					
Norvell *et al* 2019^35^	0.755 (0.688,0.822)	1.040	0.333	0.827	0.158
Kim *et al* 2021^36^	0.717 (0.666,0.769)	0.927	0.175	0.850	0.178
Campbell *et al* 2019^37^	0.646 (0.592,0.701)	0.580	0.822	0.212	0.245

MLLA revision					
Predictor	C-statistic (95% c.i.)	Calibration slope	Calibration intercept	Calibration-in-the-large	Brier score
**Healthcare professionals**					
All healthcare professionals	0.627 (0.559,0.695)	0.335	−1.361	1.560	0.151
Consultant surgeons	0.631 (0.536,0.726)	0.357	−1.350	1.479	0.136
Trainee surgeons	0.614 (0.516,0.712)	0.302	−1.370	1.647	0.169
**Outcome prediction tool**					
Czerniecki *et al* 2019^38^	0.545 (0.458,0.632)	0.147	−1.943	2.247	0.128

Ambulation					
Predictor	C-statistic (95% c.i.)	Calibration slope	Calibration intercept	Calibration-in-the-large	Brier score
**Healthcare professionals**					
All healthcare professionals	0.662 (n/a)*	n/a	n/a	n/a	n/a
Consultant surgeons	0.674 (n/a)*	n/a	n/a	n/a	n/a
Trainee surgeons	0.616 (n/a)*	n/a	n/a	n/a	n/a
Allied healthcare professionals	0.692 (n/a)*	n/a	n/a	n/a	n/a
**Outcome prediction tools**					
Czerniecki *et al* 2017^39^	0.667 (0.600,0.734)	0.318	−0.775	1.357	0.233
Bowrey *et al* 2019^40^	0.688 (n/a)*	n/a	n/a	n/a	n/a

*Multiclass area under the curve (AUC) calculated by averaging across all ‘pairwise’ AUCs. MLLA, major lower limb amputation; n/a, not applicable.

#### One-year death

**Fig. 2:**
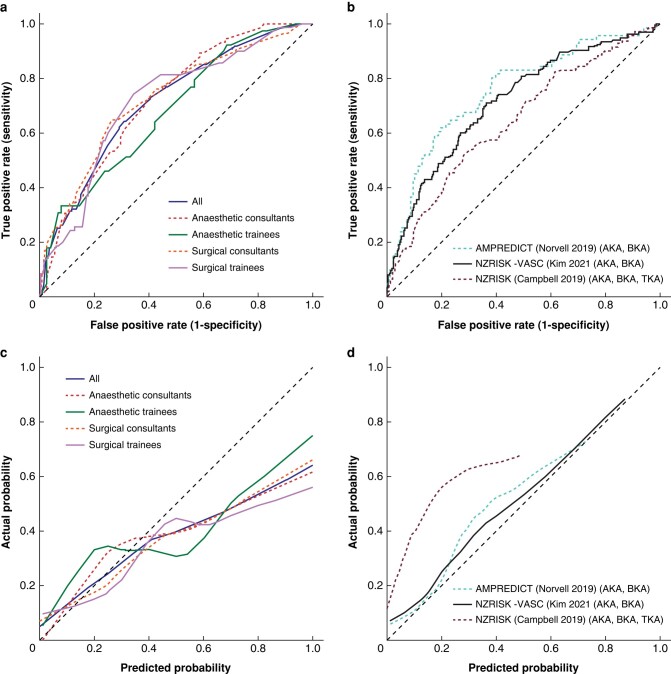
Receiver operating characteristic curves for healthcare professionals (a) and outcome prediction tools (b); and calibration curves for healthcare professionals (c) and outcome prediction tools (d) in predicting 1-year death after major lower limb amputation AKA, above knee amputation; BKA, below knee amputation; TKA, through knee amputation; NZRISK-VASC, The New Zealand Vascular Surgical Risk Tool; NZRISK, The New Zealand Surgical Risk Tool.

There were 919 recorded predictions of 1-year death risk for 482 patients with outcome data (*Fig. 2*). The number of predictions per healthcare professional group were: 316 from surgical consultants, 251 from surgical trainees, 237 from anaesthetic consultants and 115 from anaesthetic trainees.

Overall, healthcare professionals had acceptable predictive discrimination (C-statistic = 0.715, 95% c.i. 0.679 to 0.750). Overall results for calibration slope and intercept were 0.549 (range: 0.486 to 0.576) and −0.533 (range: −0.647 to −0.312). The calibration curves demonstrated a tendency for each healthcare professional group to overestimate death risk, which became more pronounced as the predicted risk increased. Calibration-in-the-large corroborated this result (overall = 1.198, range: 1.025 to 1.295). The Brier score was 0.200 (range: 0.189 to 0.220).

Three outcome prediction tools aimed to predict 1-year death^35–37^. Two had acceptable discrimination (C-statistics = 0.755, 95% c.i. 0.688 to 0.822; and 0.717, 95% c.i. 0.666 to 0.769) and were well calibrated (calibration slopes of 1.040 and 0.927; calibration intercepts of 0.333 and 0.175; calibrations-in-the-large of 0.827 and 0.850; calibration curves demonstrating only slight underestimation of risk)^35,36^; whilst one had poor discrimination (C-statistic = 0.646, 95% c.i. 0.592 to 0.701) and calibration (calibration slope = 0.580; calibration intercept = 0.822; calibration-in-the-large = 0.212; calibration curve demonstrating systematic underestimation of risk)^37^. The Brier scores ranged from 0.158 to 0.245.

#### One-year MLLA revision

**Fig. 3:**
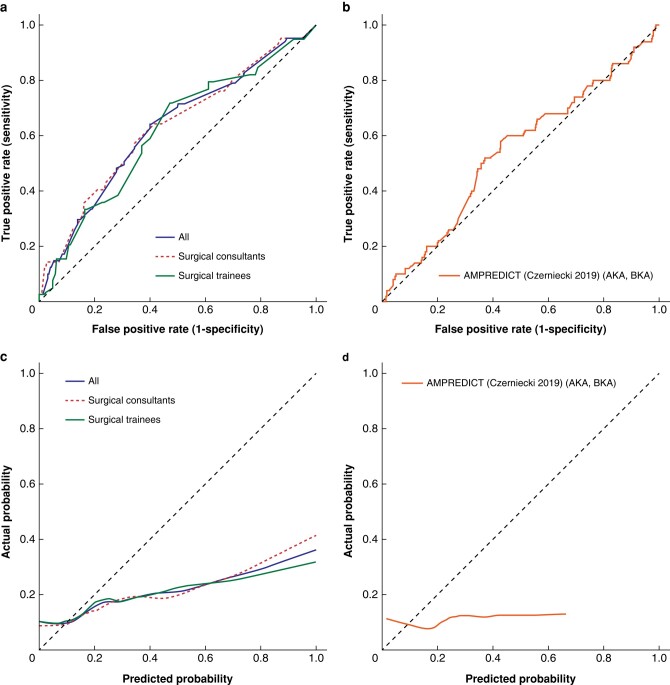
Receiver operating characteristic curves for healthcare professionals (a) and outcome prediction tools (b); and calibration curves for healthcare professionals (c) and outcome prediction tools (d) in predicting 1-year major lower limb amputation revision after major lower limb amputation AKA, above knee amputation; BKA, below knee amputation.

There were 562 recorded predictions of 1-year MLLA revision risk for 190 patients with outcome data (*Fig. 3*). The number of predictions per healthcare professional group were: 315 from surgical consultants and 247 from surgical trainees.

Surgeons had poor discrimination (C-statistic = 0.627, 95% c.i. 0.559 to 0.695; overall calibration slope = 0.335; calibration intercept = −1.361); results for consultant surgeons and trainee surgeons were similar. Surgeons tended to overestimate risk as demonstrated in the calibration curves, and by calibration-in-the-large (overall = 1.560; consultant surgeons = 1.479; trainee surgeons = 1.647). The overall Brier score was 0.151 (consultant surgeons = 0.136; trainee surgeons = 0.169).

The available outcome prediction tool had very poor discrimination (C-statistic = 0.545, 95% c.i. 0.485 to 0.632), poor calibration (calibration slope = 0.147; calibration intercept = −1.943; calibration-in-the-large = 2.247) and tended to overestimate risk^38^. The Brier score was 0.128.

#### One-year ambulation

There were 763 recorded predictions of 1-year ambulation for 489 patients with outcome data. The number of predictions per healthcare professional group were: 272 from surgical consultants, 213 from surgical trainees and 278 from allied healthcare professionals.

Overall, ambulation was poorly predicted (multiclass C-statistic = 0.662) although allied healthcare professionals performed slightly better (0.692) than consultant surgeons (0.674) and trainee surgeons (0.616). Calibration curves could not be assessed due to the outcome being multicategorical.

Two outcome prediction tools predicted 1-year ambulation^39,40^. Both had rather poor discrimination (C-statistics = 0.667 and 0.688). Calibration analyses were only possible for one tool which predicts the probability of a dichotomized outcome (SIGAM C or better) but demonstrated poor calibration (calibration slope = 0.318; calibration intercept = −0.775; calibration-in-the-large = 1.375)^39^. The Brier score was 0.233.

### Subgroup and sensitivity analyses

#### COVID-19

The performance of healthcare professionals in predicting death, MLLA revision and ambulation after excluding patients positive for COVID-19 was very similar to that of the primary analyses (*Supplementary Material S4*).

Two death predicting tools had marginally better discrimination after excluding COVID-19 patients (C-statistics = 0.773, 95% c.i. 0.704 to 0.842; and 0.753, 95% c.i. 0.697 to 0.808, compared with C-statistics = 0.755 and 0.717 in primary analyses)^35,36^. Otherwise, analyses excluding patients with COVID-19 were similar to the primary analyses.

#### Healthcare professionals using an existing outcome prediction tool

The two groups that reported using outcome prediction tools to inform their predictions frequently enough to allow for analyses were anaesthetists predicting death (*N* = 57) and allied healthcare professionals predicting ambulation (*N* = 51). The tool used most frequently by anaesthetists was the Vascular Physiological and Operative Severity Score for the enumeration of Mortality and morbidity (POSSUM)^41^. All but one of the allied healthcare professionals using an outcome prediction tool used the Blatchford Allman Russell tool (BLARt) score^40^.

**Fig. 4:**
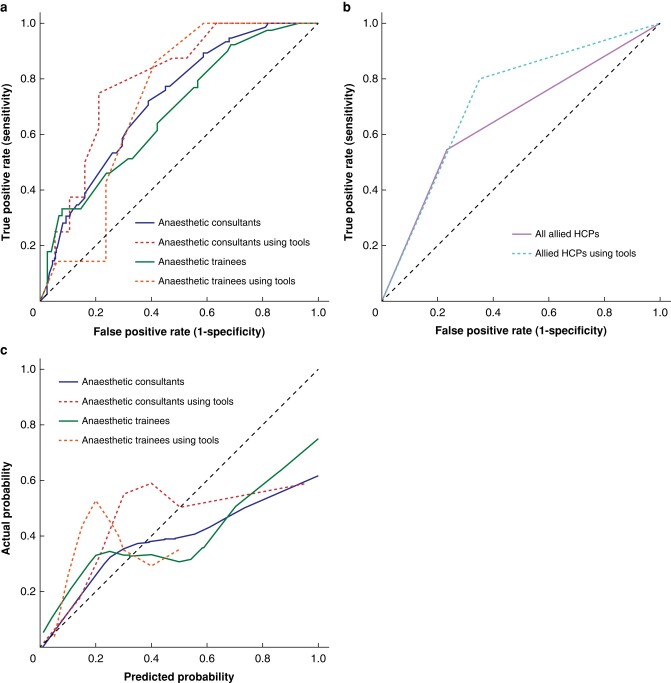
Sensitivity analyses comparing healthcare professionals (HCP) using an outcome prediction tool to inform predictions with those not using an outcome prediction tool **a** receiver operating characteristic curve for anaesthetists using/not using a tool to predict death. **b** receiver operating characteristic curve for allied healthcare professionals using/not using a tool to predict ambulation. **c** calibration curve for anaesthetists using/not using a tool to predict death.

*Figure 4* demonstrates that anaesthetists who used an outcome prediction tool routinely to predict death had marginally better discrimination (C-statistic = 0.755, 95% c.i. 0.623 to 0.887) compared with those who did not (C-statistic = 0.703, 95% c.i. 0.642 to 0.763), whilst calibration and overall performance were similar (calibration slopes = 0.528 and 0.559; calibration intercepts = −0.179 and −0.394; calibrations-in-the-large = 0.823 and 1.148; Brier scores = 0.198 and 0.216). A higher proportion of death predictions by trainee anaesthetists were informed by an existing outcome prediction tool (24/115; 20.9%) compared with consultant anaesthetists (27/237; 11.4%). Consultant anaesthetists using an outcome prediction tool had better discrimination than those who did not use a tool (0.793, 95% c.i. 0.616 to 0.970; and 0.712, 95% c.i. 0.641 to 0.783 respectively); both groups had comparable calibration and overall performance (*Supplementary Material S5*). Trainee anaesthetists who used an outcome prediction tool also had better discrimination compared with those not using an outcome prediction tool (0.718, 95% c.i. 0.514 to 0.923; and 0.680, 95% c.i. 0.564 to 0.797 respectively), but had worse calibration-in-the-large.

Allied healthcare professionals who used an outcome prediction tool to inform their ambulation predictions had marginally worse discrimination (C-statistic = 0.640) compared with those who did not (C-statistic = 0.695).

#### Other

Discrimination, calibration and overall results were similar to the primary results when sensitivity analyses based on geographical location (UK or outside UK) and statistical method to handle missing values in outcome prediction models (worst-case imputation or casewise deletion) were undertaken.

Sensitivity analyses comparing healthcare professional predictions for subgroups of patients grouped according to indication for MLLA (chronic limb-threatening ischaemia or acute limb ischaemia; diabetic complication; mixed chronic limb-threatening ischaemia and diabetic complication) are summarized in *Supplementary Material S6*. There was a tendency for more accurate death predictions and less accurate MLLA revision predictions for the diabetic complication subgroup whereas ambulation predictions were similar for all subgroups.

## Discussion

Healthcare professionals had acceptable accuracy in predicting death but were poor at predicting MLLA revision and ambulation at 1 year following MLLA. More senior (consultant level) healthcare professionals had better discrimination performance compared with trainees. Two death risk prediction tools outperformed healthcare professionals^35,36^. The only risk prediction tool that aimed to predict MLLA revision did so poorly and performed worse than healthcare professionals. The two outcome prediction tools predicting ambulation did so poorly but marginally outperformed healthcare professionals.

Healthcare professionals had acceptable discriminatory performance in predicting 1-year death, however, this was worse compared with predictions of 30-day death in the same cohort (C-statistic: 0.715 *versus* 0.758 respectively)^16^. Calibration analyses demonstrated a tendency to overestimate death risk, but to a lesser degree than predictions of 30-day death (calibration-in-the-large: 1.198 *versus* 1.908 respectively)^16^. A similar pattern (worse discrimination performance, less overestimation of risk) is also seen with predictions of MLLA revision when comparing 1-year and 30-day outcomes. These findings are in keeping with a systematic review of surgeons’ accuracy in predicting postoperative outcomes which concluded that discrimination in predicting longer-term outcomes was worse than for short-term outcomes^14^. The availability heuristic (where more easily remembered events are perceived as occurring more frequently) may partially account for these results^42–45^. The negative emotional experience of a patient requiring MLLA revision or dying early in the postoperative interval is likely more readily recalled than late postoperative events.

MLLA revision was poorly predicted. Data on surgeons’ accuracy in predicting operation-specific morbidity are scarce but predictions are generally poor^14^. MLLA revision as an outcome could be difficult to predict due to subjectivity and complexity of confounding factors. The decision to perform MLLA revision may be influenced by variability in surgical practice, since the surgeon making this decision with the patient may not be the same surgeon making the initial prediction. Similarly, unforeseen events between the primary MLLA and when an indication for MLLA revision is apparent could mean that some patients with an indication for MLLA revision may no longer be considered appropriate for operative intervention due to lack of fitness for surgery. Additionally, patients’ postoperative experience, emotional changes and quality-of-life vary^46,47^, which could influence their decision-making when MLLA revision is offered.

Factors that may influence a patient’s likelihood of ambulating following MLLA are numerous^39,48^, and research on predicting which patients will ambulate (and to which degree) is limited. This may have contributed to healthcare professionals being poor at predicting this outcome. Similarly, follow-up practices may impede learning from feedback if clinicians end follow-up once wound healing is satisfactory, which will invariably be before prosthesis fitting and rehabilitation. Allied healthcare professionals had the best discrimination in predicting ambulation, highlighting the value of multidisciplinary working. Two risk prediction tools marginally outperformed healthcare professionals’ discrimination in predicting ambulation^39,40^, but ambulation predictions were generally inaccurate.

Clinicians for whom the PERCEIVE cohort is representative of their practice should consider using one of the two outcome prediction tools that outperformed healthcare professionals in predicting 1-year death^35,36^. An outcome prediction tool that outperforms healthcare professionals is likely to complement shared decision-making; however, the clinical impact in terms of patient satisfaction with shared decision-making and decisional regret cannot be determined from this study. Ongoing qualitative research may give insight into how risk prediction tools that perform well could be used in practice with this patient group^49^. In contrast to the short-term results from the PERCEIVE study^16^, subgroup analyses revealed that anaesthetists who used a risk prediction tool routinely to inform their estimations of death had better discrimination performance, but estimations were not as well calibrated. This is possibly because the risk prediction tools used were not specific to MLLA. Conversely, in this study allied healthcare professionals who routinely used an outcome prediction tool to predict ambulation had marginally worse discrimination compared with those who did not use a tool routinely. This likely reflects ambulation being a difficult outcome to predict. Healthcare professionals, and the outcome prediction tool being used routinely by allied healthcare professionals (and evaluated in this study), performed poorly in this cohort^40^. Interestingly, when examining the results of consultant and trainee anaesthetists using/not using an outcome prediction tool separately, the trainees had much worse calibration when using an outcome prediction tool whilst results were comparable for the two consultant groups. This may be a reflection of how the results given by an outcome prediction tool are utilized—as an accurate prediction or complementary to the clinician’s intuition. Further research should explore how healthcare professionals interpret and use outcome prediction tools in practice.

The study’s strengths are that data were collected prospectively from a large number of patients and centres; results should therefore be highly relevant and applicable to the large number of clinicians for whom this cohort is representative of their practice. The evaluation of risk prediction tools included several performance metrics to give a complete picture of accuracy and our primary analyses were tested with relevant sensitivity analyses which produced similar results. The outcomes that healthcare professionals were asked to predict in this study align with the core outcome set for patients undergoing MLLA^50^, meaning they are considered amongst the most important outcomes for patients, carers and clinicians. Improving information provided to patients undergoing MLLA is a research priority for MLLA^51^.

A limitation of this study is the impact of COVID-19, which can be appreciated as the impact of an individual patient’s COVID-19 status, and the pandemic’s wider effect on healthcare systems. A positive COVID-19 status is a confounding factor that could have influenced the accuracy of both healthcare professionals and outcome prediction tools. The sensitivity analyses aimed to examine this, but do not account for the pandemic’s wider effects on healthcare systems. Recommendations from the Vascular Society of Great Britain and Ireland, for example, included the deferral of elective procedures whenever possible and consideration of a primary amputation as an alternative to complex revascularizations. Impact on other non-emergency services, such as rehabilitation services’ ability to facilitate physiotherapy and prosthesis fitting, and the change in risk/benefit considerations of admission to hospital (for MLLA revision) may have introduced bias^52^.

Other limitations include that outcomes for patients who were considered for MLLA but did not undergo amputation are unknown. Healthcare professionals were not mandated to provide predictions of outcomes for patients to be included in the study, meaning that there is a potential for participation bias when considering healthcare professionals’ predictive performance. Similarly, there is some subjectivity in the inclusion criteria for healthcare professionals (that they were sufficiently familiar with the patient and would be happy to provide estimations of outcomes in real practice), meaning generalizability is reduced. There are characteristics of the healthcare professionals (other than profession and seniority) that are unknown in this study that could confound results. Similarly, no specific training was given to healthcare professionals on how they should make their estimations (other than standardized visual analogue scales). Therefore, there is unknown heterogeneity in their method of estimating benefit and risk (for example which factors they consider more/less relevant for the outcomes), and this potentially limits the generalizability of the results. Whilst the results should be applicable to many clinicians, they may not be applicable to those based in countries that are not represented in this study.

This study confirms that there are uncertainties when trying to predict outcomes at 1 year following MLLA, especially so for MLLA revision and ambulation. This should be acknowledged and communicated to patients during shared decision-making. Different healthcare professional groups had similar accuracy in predicting relevant outcomes which supports recommendations that, when possible, the decision to undertake MLLA should be guided by a multidisciplinary team^53,54^.

## Collaborators

The Vascular and Endovascular Research Network: Aminder Singh, Athanasios Saratzis, Brenig Llwyd Gwilym, David Charles Bosanquet, George Dovell, Graeme Keith Ambler, Joseph Shalhoub, Louise Hitchman, Matthew Machin, Nikesh Dattani, Panagiota Birmpili, Rachael Forsythe, Robert Blair, Ruth Benson, Ryan Preece, Sandip Nandhra, Sarah Onida. The PERCEIVE study group: Aberdeen Royal Infirmary: Amy Campbell, Anna Celnik, Bryce Renwick, Jolene Moore, Karen Duncan, Martin Gannon, Mary Duguid, Patrice Forget. Bahrain Defence Force Hospital: Dhafer Kamal, Mahmoud Tolba, Martin Maresch, Mohamed Hatem, Mohamed Kabis. Birmingham Heartlands Hospital & Queen Elizabeth Hospital Birmingham: Ahmed Shalan, Hannah Travers, Maciej Juszczak, Mohammed Elsabbagh, Nikesh Dattani. Centro Hospitalar Sao Joao: António Pereira-Neves, João Rocha-Neves, José Teixeira. Christchurch Hospital: Eric Lim, Khaleel Hamdulay, Oliver Lyons. Countess of Chester Hospital: Ashraf Azer, Chris T Francis, Khalid Elsayed, Ragai Makar, Shady Zaki, Tamer Ghatwary-Tantawy. Derriford Hospital: Devender Mittapalli, Hashem Barakat, Jessica Taylor, Ross Melvin, Samantha Veal. General Hospital of Attica ‘KAT’: Anna Pachi, Antonia Skotsimara, Chrisostomos Maltezos, Christiana Anastasiadou, Efstratia Baili, George Kastrisios, Konstantinos Maltezos. Glenfield Hospital: Athanasios Saratzis, Badri Vijaynagar. Hairmyres Hospital: Elizabeth Montague-Johnstone, Euan Bright, Kirsty Stewart, Rahul Velineni, Simon Lau, Will King. Hippocratio Hospital: Christina Papadimitriou, Christos Karkos, Maria Mitka. Hull Royal Infirmary: Emily Chan, George Smith. Imperial College Healthcare NHS Trust: Aditya Vijay, Anita Eseenam Agbeko, Joachim Amoako, Joseph Shalhoub, Matthew Machin. Korgialenio-Benakio Hellenic Red Cross Hospital: Afroditi Antoniou, Konstantinos Roditis, Nikolaos Bessias, Paraskevi Tsiantoula, Theofanis Papas, Vasileios Papaioannou. Musgrove Park: Fiona Goodchild, George Dovell. Newcastle Freeman Hospital: Claire Dawkins, James Rammell, Sandip Nandhra. Policlinico Umberto 1 Sapienza University of Rome: Andrea Mingoli, Gioia Brachini, Paolo Sapienza, Pierfrancesco Lapolla. Queen Elizabeth University Hospital: Alan Meldrum, Keith Hussey, Lara Dearie, Manoj Nair. Queen’s Medical Centre: Andrew Duncan, Bryony Webb, Stefan Klimach. Royal Devon and Exeter: Francesca Guest, Tom Hardy. Royal Gwent Hospital: Annie Clothier, Luke Hopkins, Ummul Contractor. Royal Infirmary Edinburgh: Dominic Pang, Li En Tan, Meghan Hallatt, Olivia McBride, Rachael Forsythe. Royal Perth Bentley Group: Ben Thurston, Jacqueline Wong, Nishath Altaf, Oliver Ash. Shrewsbury Hospital: Amandeep Grewal, Matthew Popplewell, Steven Jones. Southmead Hospital: Bethany Wardle, Christopher Twine, Francesca Heigberg-Gibbons, Graeme Ambler, Kit Lam, Natalie Condie. St Thomas’ Hospital: Mustafa Musajee, Prakash Saha, Sanjay Patel, Stephen Black, Thomas Hayes. SUNY Upstate University Hospital: Ankur Chawla, Anthony Feghali, Asad Choudhry, Eric Hammond, Michael Costanza, Palma Shaw, Ronald Zerna Encalada, Scott Surowiec. University Hospital Coventry and Warwickshire: Craig Cadwallader, Philipa Clayton, Ruth Benson. University Hospital Ghent: Isabelle Van Herzeele, Lina Vermeir, Mia Geenens, Nathalie Moreels, Sybille Geers. University Hospital No.1 Collegium Medicum, Nicolaus Copernicus University: Arkadiusz Jawien, Tomasz Arentewicz. University Hospital of Heraklion: Emmanouil Tavlas, Nikolaos Kontopodis, Stella Lioudaki, Vasiliki Nyktari. University Hospital of Munster: Abdulhakin Ibrahim, Alexander Oberhuber, Jana Neu, Teresa Nierhoff. University Hospital of Patras: Konstantinos Moulakakis, Konstantinos Nikolakopoulos, Spyros Papadoulas, Stavros Kakkos. University Hospital of Trieste ASUGI: Mario D'Oria, Sandro Lepidi. University Hospital of Wales: Danielle Lowry, Frances Kent, Setthasorn Ooi. University Hospital Southampton: Benjamin Patterson, Daniel Urriza Rodriguez, Gareth F Williams, Ghadeer Hesham Elrefaey, Ibrahim Enemosah, Kamran A Gaba, Simon Williams. Waikato Hospital: Elizabeth Suthers, Manar Khashram, Odette Hart, Sinead Gormley, Stephen French. William Harvey Hospital: Hytham K S Hamid.

## Supplementary Material

zrad135_Supplementary_Data

## Data Availability

Data are not made available unless approved by the PERCEIVE study management group.
