# Supplementary material for: Long-term risk prediction after major lower limb amputation: 1-year results of the PERCEIVE study
Source: BJS Open. 2024 Jan 24;8(1):zrad135. doi: 10.1093/bjsopen/zrad135 (PMC10807997; doi:10.1093/bjsopen/zrad135)
Supplement: zrad135_Supplementary_Data [file zrad135_supplementary_data.docx]

**Long-term risk prediction after major lower limb amputation: one-year results of the PERCEIVE study**

**Authors:** Brenig Llwyd Gwilym^1^, Philip Pallmann^2^, Cherry-Ann Waldron^2^, Emma Thomas-Jones^2^, Sarah Milosevic^2^, Lucy Brookes-Howell^2^, Debbie Harris^2^, Ian Massey^3^, Jo Burton^3^, Phillippa Stewart^3^, Katie Samuel^4^, Sian Jones^5^, David Cox^5^, Annie Clothier^1^, Hayley Prout^2^, Adrian Edwards^6^, Christopher P. Twine^7^, and David Charles Bosanquet^1^ on behalf of Vascular and Endovascular Research Network (VERN) and The PERCEIVE study group

The Vascular and Endovascular Research Network: Please see separate document for list of authors

The PERCEIVE study group: Please see separate document for list of authors

**Author affiliations:**

1. Gwent Vascular Institute, Royal Gwent Hospital, Aneurin Bevan University Health Board, Newport, UK
2. Centre for Trials Research, Cardiff University, UK
3. Artificial Limb and Appliance Centre, Rookwood Hospital, Cardiff and Vale University Health Board, Cardiff, UK
4. Department of Anaesthesia, North Bristol NHS Trust, Bristol, UK
5. C/O INVOLVE Health and Care Research Wales, Cardiff, UK
6. Division of Population Medicine, Cardiff University, Cardiff, UK
7. Bristol, Bath and Weston Vascular Network, North Bristol NHS Trust, Southmead Hospital, Bristol, UK

**Corresponding authors:** Brenig Llwyd Gwilym

Address: Gwent Vascular Institute, Royal Gwent Hospital, Newport, NP20 2UB. United Kingdom

Orchid ID: 0000-0002-5403-8720

**Supplementary Materials - Index**

| **Supplementary Figures and Tables** |  |
| --- | --- |
| Supplementary material 1 | *pag. 2* |
| Supplementary material 2  Supplementary material 3  Supplementary material 4  Supplementary material 5  Supplementary material 6  Supplementary material 7 | *pag. 3*  *pag. 4*  *pag. 5*  *pag. 7*  *pag. 8*  *pag. 10* |
| **References** | *pag. 12* |
|  |  |

**Supplementary Figures and Tables**

**Supplementary material 1. PERCEIVE study cohort demographic details (adapted from Gwilym et al. 2022]**^16^**)**

| **Demographic and comorbidities** | | | |
| --- | --- | --- | --- |
| **Variable** | **N/median** | **%/range** | **Missing (%)** |
| Age | 68 | 19-94 | 0 |
| *Gender* | | | 0 |
| Male | 433 | 80.6 |  |
| Female | 104 | 19.4 |  |
| Body Mass Index | 25.9 | 13.8-52.0 | 176 (32.8) |
| *Smoking status* | | | 0 |
| Current smoker | 191 | 35.6 |  |
| Ex-smoker | 222 | 41.3 |  |
| Hypertension | 370 | 68.9 | 0 |
| Chronic obstructive pulmonary disease | 108 | 20.1 | 0 |
| Ischaemic heart disease | 214 | 39.9 | 0 |
| Chronic Heart Failure | 108 | 20.1 | 0 |
| Diabetes Mellitus | 337 | 62.8 | 0 |
| Chronic Kidney Disease | 151 | 28.1 | 2 (0.4) |
| *American Society of Anethesiologists (ASA) grade* | | | 1 (0.2) |
| ASA 1 | 1 | 0.2 |  |
| ASA 2 | 28 | 5.2 |  |
| ASA 3 | 363 | 67.6 |  |
| ASA 4 | 139 | 25.9 |  |
| ASA 5 | 5 | 0.9 |  |
| Do not resuscitate order in place | 87 | 16.2 | 3 (0.6) |
| Current sepsis | 122 | 22.7 | 0 |
| Previous ipsilateral endovascular intervention | 218 | 40.6 | 0 |
| Previous ipsilateral open vascular intervention | 141 | 26.3 | 0 |
| Previous ipsilateral minor amputation | 70 | 13.0 | 0 |
| Negative COVID-19 PCR test within 3 days preoperatively | 420 | 78.2 | 0 |
| **Procedure** | | | |
| **Variable** | **N** | **%** | **Missing (%)** |
| *Amputation level* | | | 1 (0.2) |
| Below Knee Amputation | 271 | 50.5 |  |
| Above Knee Amputation | 248 | 46.2 |  |
| Through Knee Amputation | 17 | 3.2 |  |

**Supplementary material 2. Search terms and Prisma flow diagram of the updated systematic search for outcome prediction tools that aim to predict outcomes at 1-year following MLLA.**

Search terms: Amputation [medical subject heading terms] AND (tool* OR scor*) AND (risk* OR predict* OR stratif*) AND (mortality OR morbidity OR ambulat* OR outcome* OR healing OR reamputation*)

**Identification of studies via databases and registers**

Records removed *before screening*:

Duplicate records removed: (n = 51)

Records marked as ineligible by automation tools (n = 0)

Records removed for other reasons (n = 0)

Records identified from:

Databases (n = 610)

**Identification**

Records screened:

(n = 559)

Records excluded:

(n = 556)

Reports sought for retrieval:

(n = 3)

Reports not retrieved:

(n = 0)

**Screening**

Reports assessed for eligibility:

(n = 3)

Reports excluded:

Not specific to MLLA (n = 3)

Studies included in review:

(n = 0)

**Included**

*From:*  Page MJ, McKenzie JE, Bossuyt PM, Boutron I, Hoffmann TC, Mulrow CD, et al. The PRISMA 2020 statement: an updated guideline for reporting systematic reviews. BMJ 2021;372:n71. doi: 10.1136/bmj.n71

**Supplementary material 3. Variables required for each of the outcome prediction tools.**

| **First author and year** | **Outcome predicted** | **Variables in the regression equation** |
| --- | --- | --- |
| Bowrey 2019 | Ambulation | Sex, age, body mass index, functional status, severe respiratory disease, dialysis, stroke/neurological disease, myocardial infarction (last 6 months), claudication, ulcers, knee-replacement, previous amputation, indication for amputation, level of amputation, bilateral case, ability to understand and retain information, alert |
| Campbell 2019 | Mortality | Age, sex, emergency, American Society of Anesthesiologists classification, cancer, surgical grade, ethnicity, vascular specialty |
| Czerniecki 2016 | Ambulation | Amputation level, age, body mass index, race, marital status, education, diabetes, dialysis, chronic obstructive pulmonary disease, anxiety/depression, self-rated health |
| Czerniecki 2019 | MLLA revision | Amputation level, sex, smoking status, alcohol abuse, rest pain/gangrene, anticoagulant use, diabetes, previous revascularisation, chronic obstructive pulmonary disease, white blood cell count |
| Kim 2021 | Mortality | Age, sex, emergency, American Society of Anesthesiologists classification, cancer, diabetes, acute kidney injury, chronic kidney disease, dialysis |
| Norvell 2019 | Mortality | Amputation level, age, body mass index, race, functional status, chronic heart failure, dialysis, blood urea nitrogen, white blood cell count, platelet count |

**Supplementary material 4. Performance metrics of healthcare professionals and outcome prediction tools in sensitivity analyses (excluding COVID-19 positive patients), in predicting mortality, MLLA revision, and ambulation 1-year after MLLA.**

| **Mortality** | | | | | |
| --- | --- | --- | --- | --- | --- |
| **Predictor** | **C-statistic (95% confidence interval)** | **Calibration slope** | **Calibration intercept** | **Calibration-in-the-large** | **Brier score** |
| Healthcare professionals | | | | | |
| All healthcare professionals | 0.715 (0.679-0.750) | 0.549 | -0.533 | 1.198 | 0.200 |
| All excluding COVID | 0.709 (0.669-0.749) | 0.523 | -0.694 | 1.302 | 0.196 |
| Outcome prediction tools | | | | | |
| Norvell 2019 | 0.755 (0.688-0.822) | 1.040 | 0.333 | 0.827 | 0.158 |
| Norvell 2019 excluding COVID | 0.773 (0.704-0.842) | 1.104 | 0.291 | 0.894 | 0.148 |
| Kim 2021 | 0.717 (0.666-0.769) | 0.927 | 0.175 | 0.850 | 0.178 |
| Kim 2021 excluding COVID | 0.753 (0.697-0.808) | 1.090 | 0.178 | 0.946 | 0.159 |
| Campbell 2019 | 0.646 (0.592-0.701) | 0.580 | 0.822 | 0.212 | 0.245 |
| Campbell 2019 excluding COVID | 0.660 (0.598-0.721) | 0.604 | 0.754 | 0.238 | 0.221 |
| **MLLA revision** | | | | | |
| **Predictor** | **C-statistic (95% confidence interval)** | **Calibration slope** | **Calibration intercept** | **Calibration-in-the-large** | **Brier score** |
| Healthcare professionals | | | | | |
| All healthcare professionals | 0.627 (0.559-0.695) | 0.335 | -1.361 | 1.560 | 0.151 |
| All excluding COVID | 0.621 (0.545-0.696) | 0.350 | -1.308 | 1.527 | 0.153 |
| Outcome prediction tools | | | | | |
| Czerniecki 2019 | 0.545 (0.458-0.632) | 0.147 | -1.943 | 2.247 | 0.128 |
| Czerniecki 2019 excluding COVID | 0.559 (0.469-0.648) | 0.205 | -1.808 | 2.110 | 0.129 |
| **Ambulation** | | | | | |
| **Predictor** | **C-statistic (95% confidence interval)** | **Calibration slope** | **Calibration intercept** | **Calibration-in-the-large** | **Brier score** |
| Healthcare professionals | | | | | |
| All healthcare professionals | 0.662 (n/a)* | n/a | n/a | n/a | n/a |
| All excluding COVID | 0.652 (n/a)* | n/a | n/a | n/a | n/a |
| Outcome prediction tools | | | | | |
| Czerniecki 2016 | 0.667 (0.600-0.734) (n/a) | 0.318 | -0.775 | 1.357 | 0.233 |
| Czerniecki 2016 excluding COVID | 0.656 (0.583, 0.729) | 0.293 | -0.779 | 1.359 | 0.241 |
| Bowrey 2019 | 0.688 (n/a)* | n/a | n/a | n/a | n/a |
| Bowrey 2019 excluding COVID | 0.682 (n/a)* | n/a | n/a | n/a | n/a |

* Multi-class AUC calculated by averaging across all ‘pairwise’ AUCs

**Supplementary material 5. Performance metrics of Anaesthetists in predicting mortality, comparison of those who used an outcome prediction tool with those who did not.**

| **Mortality** | | | | | |
| --- | --- | --- | --- | --- | --- |
| **Predictor** | **C-statistic** | **Calibration slope** | **Calibration intercept** | **Calibration-in-the-large** | **Brier score** |
| Healthcare professionals | | | | | |
| Consultant Anaesthetists using an outcome prediction tool | 0.793 | 0.498 | -0.409 | 1.015 | 0.190 |
| Consultant Anaesthetists not using an outcome prediction tool | 0.712 | 0.593 | -0.397 | 1.163 | 0.213 |
| Trainee Anaesthetists using an outcome prediction tool | 0.718 | 0.821 | 0.505 | 0.603 | 0.206 |
| Trainee Anaesthetists not using an outcome prediction tool | 0.680 | 0.488 | -0.381 | 1.117 | 0.224 |

**Supplementary material 6. Sensitivity analyses comparing healthcare professionals’ performance in predicting mortality, MLLA revision, and ambulation for subgroups based on indication for major lower limb amputation.**

| **Chronic limb threatening ischaemia or acute limb ischaemia** | | | | | | |
| --- | --- | --- | --- | --- | --- | --- |
| **Outcome** | | **C-statistic (95% confidence interval)** | **Calibration slope** | **Calibration intercept** | **Calibration-in-the-large** | **Brier score** |
| 1-year mortality | | 0.743 (0.696-0.790) | 0.606 | -0.465 | 1.165 | 0.192 |
| 1-year MLLA revision | | 0.627 (0.544-0.709) | 0.310 | -0.969 | 1.078 | 0.167 |
| 1-year ambulation | | 0.646 (n/a)* | n/a | n/a | n/a | n/a |
|  |  | | | | | |
| **Diabetic complication** | | | | | | |
| **Outcome** | | **C-statistic (95% confidence interval)** | **Calibration slope** | **Calibration intercept** | **Calibration-in-the-large** | **Brier score** |
| 1-year mortality | | 0.625 (0.511-0.739) | 0.345 | -1.377 | 2.114 | 0.221 |
| 1-year MLLA revision | | 0.803 (0.696-0.909) | 0.753 | -2.167 | 4.243 | 0.143 |
| 1-year ambulation | | 0.687 (n/a)* | n/a | n/a | n/a | n/a |
|  |  | | | | | |
| **Mixed chronic limb threatening ischaemia and diabetic complication** | | | | | | |
| **Outcome** | | **C-statistic (95% confidence interval)** | **Calibration slope** | **Calibration intercept** | **Calibration-in-the-large** | **Brier score** |
| 1-year mortality | | 0.717 (0.655-0.779) | 0.626 | -0.191 | 1.001 | 0.202 |
| 1-year MLLA revision | | 0.638 (0.481-0.796) | 0.503 | -1.667 | 2.131 | 0.126 |
| 1-year ambulation | | 0.671 (n/a)* | n/a | n/a | n/a | n/a |

* Multi-class AUC calculated by averaging across all ‘pairwise’ AUCs

**Supplementary material 7. List of collaborative co-authors**

The Vascular and Endovascular Research Network (Alphabetical order):

Graeme Keith Ambler, Ruth Benson, Panagiota Birmpili, Robert Blair, David Charles Bosanquet, Nikesh Dattani, George Dovell, Rachael Forsythe, Brenig Llwyd Gwilym, Louise Hitchman, Matthew Machin, Sandip Nandhra, Sarah Onida, Ryan Preece, Athanasios Saratzis, Joseph Shalhoub, Aminder Singh.

The PERCEIVE study group (Centres in alphabetical order):

Aberdeen Royal Infirmary: Patrice Forget, Martin Gannon, Anna Celnik, Mary Duguid, Amy Campbell, Karen Duncan, Bryce Renwick, Jolene Moore. Bahrain Defence Force Hospital: Martin Maresch, Mahmoud Tolba, Dhafer Kamal, Mohamed Kabis, Mohamed Hatem. Birmingham Heartlands Hospital & Queen Elizabeth Hospital Birmingham: Maciej Juszczak, Nikesh Dattani, Hannah Travers, Ahmed Shalan, Mohammed Elsabbagh. Centro Hospitalar Sao Joao: João Rocha-Neves, António Pereira-Neves, José Teixeira. Christchurch Hospital: Oliver Lyons, Eric Lim, Khaleel Hamdulay. Countess of Chester Hospital: Ragai Makar, Shady Zaki, Chris T Francis, Ashraf Azer, Tamer Ghatwary-Tantawy, Khalid Elsayed. Derriford Hospital: Devender Mittapalli, Ross Melvin, Hashem Barakat, Jessica Taylor, Samantha Veal. William Harvey Hospital: Hytham K S Hamid. General Hospital of Attica ‘KAT’: Efstratia Baili, George Kastrisios, Chrisostomos Maltezos, Konstantinos Maltezos, Christiana Anastasiadou, Anna Pachi, Antonia Skotsimara. Glenfield Hospital: Athanasios Saratzis, Badri Vijaynagar. Hairmyres Hospital: Simon Lau, Rahul Velineni, Euan Bright, Elizabeth Montague-Johnstone, Kirsty Stewart, Will King. Hippocratio Hospital: Christos Karkos, Maria Mitka, Christina Papadimitriou. Hull Royal Infirmary: George Smith, Emily Chan. Imperial College Healthcare NHS Trust: Joseph Shalhoub, Matthew Machin, Anita Eseenam Agbeko, Joachim Amoako, Aditya Vijay. Korgialenio-Benakio Hellenic Red Cross Hospital: Konstantinos Roditis, Vasileios Papaioannou, Afroditi Antoniou, Paraskevi Tsiantoula, Nikolaos Bessias, Theofanis Papas. Musgrove Park: George Dovell, Fiona Goodchild. Newcastle Freeman Hospital: Sandip Nandhra, James Rammell, Claire Dawkins. Policlinico Umberto 1 Sapienza University of Rome: Pierfrancesco Lapolla, Paolo Sapienza, Gioia Brachini, Andrea Mingoli. Queen Elizabeth University Hospital: Keith Hussey, Alan Meldrum, Lara Dearie, Manoj Nair. Queen’s Medical Centre: Andrew Duncan, Bryony Webb, Stefan Klimach. Royal Devon and Exeter: Tom Hardy, Francesca Guest. Royal Gwent Hospital: Luke Hopkins, Ummul Contractor, Annie Clothier. Royal Infirmary Edinburgh: Olivia McBride, Meghan Hallatt, Rachael Forsythe, Dominic Pang, Li En Tan. Royal Perth Bentley Group: Nishath Altaf, Jacqueline Wong, Ben Thurston, Oliver Ash. Shrewsbury Hospital: Matthew Popplewell, Amandeep Grewal, Steven Jones. Southmead Hospital: Bethany Wardle, Christopher Twine, Graeme Ambler, Natalie Condie, Kit Lam, Francesca Heigberg-Gibbons. St Thomas’ Hospital: Prakash Saha, Thomas Hayes, Sanjay Patel, Stephen Black, Mustafa Musajee. SUNY Upstate University Hospital: Asad Choudhry, Eric Hammond, Michael Costanza, Palma Shaw, Anthony Feghali, Ankur Chawla, Scott Surowiec, Ronald Zerna Encalada. University Hospital Coventry and Warwickshire: Ruth Benson, Craig Cadwallader, Philipa Clayton. University Hospital Ghent: Isabelle Van Herzeele, Mia Geenens, Lina Vermeir, Nathalie Moreels, Sybille Geers. University Hospital No.1 Collegium Medicum, Nicolaus Copernicus University: Arkadiusz Jawien, Tomasz Arentewicz. University Hospital of Heraklion: Nikolaos Kontopodis, Stella Lioudaki, Emmanouil Tavlas, Vasiliki Nyktari. University Hospital of Munster: Alexander Oberhuber, Abdulhakin Ibrahim, Jana Neu, Teresa Nierhoff. University Hospital of Patras: Konstantinos Moulakakis, Stavros Kakkos, Konstantinos Nikolakopoulos, Spyros Papadoulas. University Hospital of Trieste ASUGI: Mario D'Oria, Sandro Lepidi. University Hospital of Wales: Frances Kent, Danielle Lowry, Setthasorn Ooi. University Hospital Southampton: Ibrahim Enemosah, Benjamin Patterson, Simon Williams, Ghadeer Hesham Elrefaey, Kamran A Gaba, Gareth F Williams, Daniel Urriza Rodriguez. Waikato Hospital: Manar Khashram, Sinead Gormley, Odette Hart, Elizabeth Suthers, Stephen French.

**References**

**Supplementary material 2:**

Page MJ, McKenzie JE, Bossuyt PM, Boutron I, Hoffmann TC, Mulrow CD, et al. The PRISMA 2020 statement: an updated guideline for reporting systematic reviews. BMJ 2021;372:n71. doi: 10.1136/bmj.n71
